# A Casual Video Game With Psychological Well-being Concepts for Young Adolescents: Protocol for an Acceptability and Feasibility Study

**DOI:** 10.2196/31588

**Published:** 2021-08-12

**Authors:** Russell Pine, James Mbinta, Lisa Te Morenga, Theresa Fleming

**Affiliations:** 1 School of Health Victoria University of Wellington Wellington New Zealand; 2 Research Centre for Hauora and Health Massey University Wellington New Zealand

**Keywords:** digital mental health tools, casual video games, young people

## Abstract

**Background:**

Many face-to-face and digital therapeutic supports are designed for adolescents experiencing high levels of psychological distress. However, promoting psychological well-being among adolescents is often neglected despite significant short-term and long-term benefits.

**Objective:**

This research has 3 main objectives: (1) to assess the acceptability of Match Emoji, a casual video game with psychological well-being concepts among 13-15-year-old students in a New Zealand secondary school; (2) to identify the feasibility of the research process; and (3) to explore the preliminary well-being and therapeutic potential of Match Emoji.

**Methods:**

Approximately 40 participants aged 13-15 years from a local secondary college in Wellington, New Zealand, will be invited to download and play Match Emoji 3-4 times a week for 5-15 minutes over a 2-week period. Participants will complete 4 assessments at baseline, postintervention, and 3 weeks later to assess psychological well-being and therapeutic changes. Statistical analysis will be used to synthesize data from interviews and triangulated with assessment changes and game analytics. This synthesis will help to assess the acceptability and feasibility of the Match Emoji.

**Results:**

The key outputs from the project will include the acceptability, feasibility, and therapeutic potential of Match Emoji. It is anticipated that participants will have finished playing the recommended game play regimen by August 2021 with analysis of results completed by October 2021.

**Conclusions:**

Data from the study are expected to inform future research on Match Emoji including a randomized controlled trial and further adjustments to the design and development of the game.

**International Registered Report Identifier (IRRID):**

PRR1-10.2196/31588

## Introduction

In New Zealand, an increasing number of young people experience elevated levels of psychological distress and low well-being [1]. Although treatments such as cognitive behavioral therapy exist and have shown promise for reducing clinical levels of psychological distress [2,3], supports for promoting psychological well-being are often underutilized despite their therapeutic potential [4-6].

Young adolescents, aged between 13 and 15 years, are particularly vulnerable to experiencing elevated levels of psychological distress and low well-being [7,8]. This is in part due to the additional external and internal demands placed on young adolescents from navigating puberty to the formation of gender norms and health and well-being attitudes [9,10]. To compound rapid developmental changes, young adolescents enter a more complex educational environment while forging new relationships with peers and family members. As approximately half of all mental ill-health starts by age 15 years and 75% develops by age 18 years [7], it is vital to create interventions that will promote psychological well-being skills among young adolescents.

Young adolescents in New Zealand who have access to the curriculum are provided with opportunities to learn mental health and well-being skills such as stress management and resilience skills [11,12]. Although education through the curriculum is a promising preventive measure [12,13], longitudinal research suggests more targeted supports are required to promote psychological well-being among young adolescents [14].

Given the popularity of digital technologies, a plethora of digital mental health interventions (DMHIs) have been created and shown promise for alleviating psychological distress and promoting psychological well-being in trials [15-17]. This scalable and low-cost approach is promising for young people, considering the potential to bypass traditional barriers such as stigma and time [18-20]. Recent systematic reviews and meta-analyses, however, report DMHIs are yet to reach their full engagement potential, with low real-world use of many popular mental health apps [17,21].

A growing amount of research has attempted to identify ways in which to increase adherence to DMHIs among young people [17,22]. One promising approach is using microinterventions. The goal of microinterventions is to enable users to work towards a highly focused goal with support from in-the-moment elements such as reminders and nudges [23].

A popular activity among many young people that utilizes similar underlying mechanics of microinterventions are casual video games (CVGs). Globally, CVGs such as “Bejewelled” and “Angry Birds” are played by millions of people in short bursts of time [24]. According to a recent systematic review of the literature, CVGs may also hold promising therapeutic mood enhancing and brief releases from unpleasant experiences [25]. Previous research with young adolescents suggests CVGs are a popular approach among this age group who commonly play these games to distract and “calm a busy mind” [26].

Based upon a systematic review of the literature and research with young adolescents, we created Match Emoji, a CVG with psychological well-being concepts for young adolescents. Although it is important to evaluate core psychotherapeutic components of interventions to understand how specific elements guide the design of the intervention as a whole [27], it is more useful to investigate the potential for real-world usage in naturalistic settings [21,28], As such, the aim of the current protocol is: (1) to assess the acceptability of Match Emoji among 13-15-year old students in a New Zealand secondary school, (2) to identify the feasibility of the research process, and (3) to explore the preliminary well-being and therapeutic potential of Match Emoji.

## Methods

### Research Strategy

This study will employ a mixed methods design to assess the acceptability, feasibility, and therapeutic potential of a CVG with psychological well-being concepts among 13-15-year-old students.

### Study Design

The study will involve 3 phases. First, we will recruit 13-15-year-old students from 2-4 classrooms in a local secondary school within the Wellington region of New Zealand. Once participants have returned their consent and assent forms, they will be asked to download Match Emoji onto their phone or digital device. During the second phase, participants will be encouraged to play the game 3-4 times a week for 5-15 minutes for a 2-week period. Game play time and sessions will be collected through the Unity platform to help inform the feasibility and acceptability of the recommended game play. During the third phase, researchers will follow up 2 weeks after the recommended regimen of Match Emoji and collect secondary outcomes measures (from the Child and Adolescent Mindfulness Measure [CAMM], General Help-Seeking Questionnaire [GHSQ], Flourishing Scale [FS], and Revised Children's Anxiety and Depression Scale [RCADS]), followed by short interviews with participants about their experience.

### Study Population

Approximately 40 students from a local secondary school in Wellington, New Zealand, will take part in playing Match Emoji 3-4 times a week for 5-15 minutes over a 2-week period. Participants will be invited from year 9 and 10 classrooms and are typically between 13 and 15 years of age. As this is an acceptability and feasibility study, a total of 40 participants will provide a large enough sample size to show a meaningful difference in the primary and secondary outcomes between baseline, postintervention, and a 3-week follow-up.

### Inclusion Criteria

Young people will be included in the study if they are between the ages of 13 and 15 years, have provided written consent from a parent or caregiver, and are able to understand and sign the assent form.

### Exclusion and Safety Criteria

Young people will be excluded from participation if they do not meet the inclusion criteria. The appropriate personnel within the secondary school will be engaged if a participant self-reports a high level of mental health need. This will be determined through the 4 questionnaires. Those participants who are engaged with existing therapeutic support are able to participate in the research study if consent has been obtained from the young person.

### Intervention

**Figure 1:**
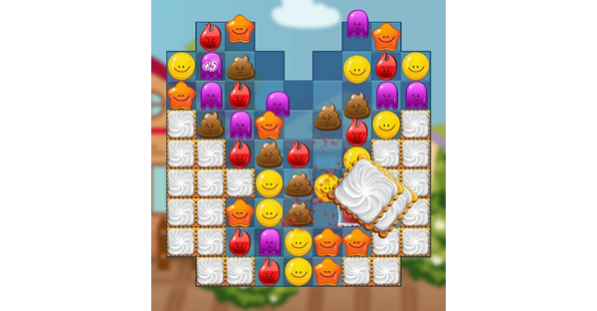
Image of the Match Emoji game.

Match Emoji is a match-3 CVG with psychological well-being concepts designed for young adolescents. The aim of Match Emoji is to match similar colored emojis together to earn points and progress through the game. There is a total of 6 different colored and shaped emojis that represent a unique digital expression such as an emotion, idea, or personality. When the user has successfully matched the required number of emojis with a fixed number of moves or time frame, a micromessage appears on the screen. Each micromessage consists of a short psychological well-being concept such as “notice what is going on around you” or “sometimes talking to a friend can help” and is delivered via a dynamic messaging loading system that identifies the “optimal” time to display the message. Hints are used throughout the game if players get stuck. For example, if the player waits too long before making a move, Match Emoji identifies a potential combination of similar colored emojis by moving a successful sequence of items back and forth to capture the user's attention. An example of the game can be seen in Figure 1.

### Outcome Measures

The primary outcomes of the study are (1) acceptability of Match Emoji (ie, is Match Emoji acceptable among young adolescents), as assessed via a short semistructured intervention with participants after the recommended regime of game play and game analytics including the number of sessions and minutes played recorded via the Unity platform, and (2) feasibility of Match Emoji (ie, is it easy to complete the study with young adolescents within a secondary school context) as measured by the number of students interested in participating in completing the 4 questionnaires, playing the recommended regimen of Match Emoji, and attending the follow-up interview.

The secondary outcome (measured at baseline before accessing Match Emoji, on completing the 2 weeks of recommended game play, and at the 3-week follow-up) is efficacy (ie, does Match Emoji promote psychological well-being skills). This will be assessed by measuring changes over time in the CAMM, GHSQ, FS, and RCADS. It is estimated to take participants approximately 10-15 minutes to complete all questionnaires.

The CAMM is a 10-item measure of mindfulness for use with children and adolescents and has been reported to have good internal consistency and significant correlations between CAMM scores and measures of psychological functioning and distress [29]. The GHSQ is a 1-page questionnaire with 2 sets of questions that examine the respondent’s likelihood of seeking help for a specific issue such as psychological distress. The GHSQ has been reported to have good reliability and validity and appears to be a flexible measure of help-seeking intentions that can be applied to different contexts and age groups including young people [30].

The 8-item FS is a valid and reliable brief summary measure of psychological well-being suited for young people [31,32]. The scale provides a single psychological well-being score derived from the 8 questions and has been used as an effective measure to access adolescents’ psychological well-being in the New Zealand secondary school context [33]. The RCADS is a youth self-report questionnaire with 6 subscales including separation anxiety disorder and low mood. The RCADS has good reliability on subscales and total scale [34], internal consistency, and good convergent validity [35]. The RCADS has been used as an appropriate and easy-to-administer assessment tool of anxiety and depressive symptoms in several populations within New Zealand [35,36].

Interviews lasting approximately 30 minutes will take place with no more than 6 participants at one time to understand experiences with playing Match Emoji. Interviews will be conducted by the first author (RP) at the local school in a setting familiar to the participants. Responses will be recorded in a paper-based format. Questions will involve (1) What parts of the game did you like? (2) What parts of the game could be improved? (3) What did you learn from playing the game? (4) Did you try and use any of the ideas from the game and if so, which ones? (5) Do you think you will continue to play Match Emoji? Interviews will not involve more than 6 participants at a time. At the end of the interview, participants will be able to read and correct answers.

### Statistical Analysis

Quantitative data from the 4 assessments and game play usage will be analyzed using Microsoft Excel, SPSS version 26, and the metrics recorded from the Unity platform including number of sessions and minutes played [37,38]. Analyses will include descriptive statistics (eg, number of sessions completed, number of minutes played, changes in assessment scores, and sociodemographic characteristics of the participants).

As this is an acceptability and feasibility study, a sample size of 40 participants will be a large enough sample to show a meaningful difference in primary and secondary outcomes between baseline and the end of the interview. Chi-square tests and *t* tests will be used to assess the statistical significance of changes in the 4 assessment scores over time. A *P* value <.05 will be used at the 95% confidence level to determine the therapeutic potential of any difference between pre- and postmeasures. NVivo will be used to store and code qualitative data from the interviews with participants. A general inductive approach will be used by researchers to identify and analyze emerging themes [39].

### Ethics and Consent

This study received ethics approval from the New Zealand Health and Disability Ethics Committee (21/NTA/34) on May 28, 2021. After the college principal or senior management staff member has understood and approved the research, participants will be provided with information about the study. Students will be provided with time to ask questions before deciding to provide informed decision about their voluntary participation through an assent form. A consent form will also be required from the parent or guardian.

All the project data and materials sent for publication will be de-identified by removing statements identifying participants. Participants who disclose mental health needs that meet the threshold for a clinical diagnosis will be handled by appropriate school personnel such as a school counsellor. The data will be stored securely in a password-protected computer accessible only to the research team. The de-identifiable findings will be included in the first author’s (RP) doctoral thesis as well as being disseminated through peer-reviewed academic journals, national and international conferences, and public events. If parents ask for their child’s individual results such as game analytics, we will seek permission from the child first.

## Results

Recruitment of participants started in June 2021, with completion anticipated to be completed by July 2021. It is anticipated that participants will have finished playing the recommended game play regimen by August 2021 with analysis of results completed by October 2021. The key outputs from the game will inform future design and iterations of the game. In addition, a larger and more robust methodological approach such as a randomized controlled trial may be created to fully understand the therapeutic effects of Match Emoji.

## Discussion

Promoting psychological well-being among young adolescents may support overall health and improve disease-specific outcomes later in life [40-42]. Given the potential benefits of promoting psychological well-being coupled with a heightened risk of experiencing elevated levels of psychological distress, it is crucial to explore engaging, preventive tools for young adolescents [43]. This is particularly important in New Zealand where a growing number of young people have reported experiencing psychological distress [14].

The current acceptability and feasibility study aims to assess the acceptability of Match Emoji among 13-15-year-old students in a New Zealand secondary school, identify the feasibility of the research process, and examine the psychological well-being and therapeutic potential of the game. The primary outcomes of the study will help to shape the iterative design process of Match Emoji and understand if the game is worthy of more rigorous testing in a randomized controlled trial. The secondary outcomes will examine the psychological well-being and therapeutic potential of Match Emoji. If Match Emoji is shown in subsequent studies to be acceptable and useful for young adolescents in its final form, it is hoped that the game may be promoted and available free of charge to young people in New Zealand on Google Play and App Store.

## Appendix

Multimedia Appendix 1 Original peer-review report from the funding agency (Health and Disability Ethics Committees, Ministry of Health, New Zealand).

Multimedia Appendix 2 Peer review from academic.

## Abbreviations

CAMM: Child and Adolescent Mindfulness Measure
CVG: casual video game
DMHI: digital mental health intervention
FS: Flourishing Scale
GHSQ: General Help-Seeking Questionnaire
RCADS: Revised Children's Anxiety and Depression Scale
