# Supplementary material for: A Casual Video Game With Psychological Well-being Concepts for Young Adolescents: Protocol for an Acceptability and Feasibility Study
Source: JMIR Res Protoc. 2021 Aug 12;10(8):e31588. doi: 10.2196/31588 (PMC8391754; doi:10.2196/31588)
Supplement: Multimedia Appendix 1 [file resprot_v10i8e31588_app1.pdf]

Health and Disability Ethics Committees

Health and Disability Ethics Committees
Ministry of Health
133 Molesworth Street
PO Box 5013
Wellington
6011

0800 4 ETHICS
hdecs@health.govt.nz

06 April 2021

Mr Russell Pine
Kelburn, Wellington 6012
Wellington 6011

Dear Mr Pine

Re:	**Ethics ref:**	**21/NTA/34**
	Study title:	The Feasibility and Acceptability of a Casual Video Game with Mental Wellbeing Concepts Among Year 9 and 10 Students.

This application was reviewed by the Northern A Health and Disability Ethics Committee and *provisionally approved* pending receipt of further information. This decision was made through the HDEC-Full Review pathway.

Summary of Study

1. The Feasibility and Acceptability of a Casual Video Game with Mental Wellbeing Concepts Among Year 9 and 10 Students. Classroom based recruitment (n=40), game played a few times a week, pre-post pysch/wellbeing measures and game play analytics.

Summary of resolved ethical issues

The main ethical issues considered by the Committee and addressed by the Researcher are as follows.

2. The Committee considered if it was appropriate for the 13-15-year-old participants to consent for themselves rather than gaining consent of parents. The consensus of the Committee (with one member disagreeing) was that is appropriate for the parents to consent and the participants to assent.
3. The Committee asked how any mental health issues identified from the questionnaire results would be followed up. The researcher stated that these participants would be referred to the school counsellor/support, as per usual school process, who have the appropriate skills and process to assess the individual’s needs, and would consult with parents and agree a joint referral to a health professional where required. The Committee was comfortable with this approach and recommended that it is made clear to the participants upfront of this potential procedure.
4. The Committee acknowledged that a data management plan has been developed for this study, however it was uploaded after the agenda cut-off date and has not been reviewed by the Committee.

Summary of outstanding ethical issues

The main ethical issues considered by the Committee and which require addressing by the Researcher are as follows.

5. The Committee asked for clarity on where the participants' data analytics for the game will be captured, who will have access to it and how the privacy of participants will be safeguarded. The researcher confirmed that the game is on the Unity platform and will not be downloaded using an app store (contrary to the uploaded documentation). The researcher advised that they are working through a more secure approach to keeping the participants' data unidentifiable to third parties. The approach the researchers now intend to adopt is to download the game to the university database where the participants can access it via a link rather than downloading it individually themselves. Once the participant has accessed the game from the link, they will be given a unique study number, by the researchers, that can be matched to their data usage. The Committee were comfortable with this change in approach to protecting participant's privacy and requested this change is reflected in the study documentation. Please update the protocol with more information on the Unity platform, the privacy safeguards and the role of any other third party that will have access to the identifiable data.

6. The Committee advised of the requirement to provide an independent peer review for the study protocol. (National Ethical Standards for Health and Disability Research and Quality Improvement, para 9.26). Please provide an independent peer review using the template and guidance on the HDEC website - https://ethics.health.govt.nz/guides-templates-forms-0/scientific-peer-review-submissions-%E2%80%93-guidance

7. The Committee requested that the protocol and participant information sheets are updated to include the following information about the study;
   a) Detailed information about the game
   b) Where and when the interviews will be conducted, including how the confidentiality of participants will be protected, whether participants can read and correct answers.
   c) Explanation of the cultural aspects of the study, if applicable
   d) The Committee suggested uploading a CV for one of the supervisors involved in the study.

8. The Committee noted that the researcher verbally advised that as well as doing a presentation to year 9 and 10 students, they may also hold a similar event for parents. The Committee agreed this was a good idea and recommended that the researcher ensure the procedures and documentation are completed first. This will enable them to use participant information sheets as a script to ensure the information that is being shared in these forums is accurate and consistent. The Committed requested that the detail on the presentations that both the students and parents will be receiving is provided to them for review.

9. The Committee stated that confidentiality may be slightly more difficult to protect in this study because the kids at school are likely to know who is participating in the research (depending upon who turns up to the presentation and where the interviews will be conducted). The Committee requested that this is made clear to the participants and parents.

<u>The Committee requested the following changes to the Participant Information Sheet and Consent Form:</u>

10. The Committee suggested that the Researchers use the HDEC participant information sheet template (https://ethics.health.govt.nz/guides-templates-forms-0/participant-information-sheet-templates). In particular, it is important to expand on the information concerning the management of participants' data, and risks

11. Provide more information about the nature of the game.

12. Include where and when the interviews will be conducted and how the confidentiality of participants will be protected, and whether participants can read and correct answers.
13. Provide detail on the presentation that both the students and parents will be receiving.
14. Please make it clear that the completion of the surveys or use of the tool will not result in a medical diagnosis and will not result in treatment.
15. Please outline the mental health referral pathway.
16. Proofread all documentation for spelling and grammatical errors (e.g. replace occurrences of “ascent” with “assent”).
17. PISs should state whether Match Emoji is free.
18. Please correct the statements in the PISs that only the researchers will know the participants names as this is not accurate.
19. Please give the Youthline and other help numbers greater prominence on the forms.
20. Please transfer the confidentiality rider from the footnote to the main body.
21. Data must be kept for longer than five years, please amend this in the forms.

Further information requested

The further information requested in order for the Northern A Health and Disability Ethics Committee to make a final decision is as follows.

22. Please address all outstanding ethical issues, providing the information requested by the Committee.
23. Please update the various information sheets and consent/assent forms, taking into account feedback provided by the Committee. *(National Ethical Standards for Health and Disability Research and Quality Improvement, para 7.15 – 7.17).*
24. Please supply an independent peer review for the current version of the study protocol. *(National Ethical Standards for Health and Disability Research and Quality Improvement, para 9.26).*
25. Please update the study protocol, taking into account the feedback provided by the Committee. *(National Ethical Standards for Health and Disability Research and Quality Improvement, para 9.7).*
26. Please note that the Data Management Plan, not reviewed by the Committee at the time of its meeting, will be considered and comments made on it, including requiring possible amendments if the Plan does not comply with the *National Ethical Standards for Health and Disability Research and Quality Improvement*, *para 12.15)*

Responding to requests for further information

In addition to making requested changes to study documentation, a cover letter should be used to respond to any outstanding ethical concerns.

As the application form cannot be edited when responding to a provisional approval, please address any concerns raised about questions in the application form in a cover letter.

Please remember to track or highlight changes made to new versions of existing documentation. Both tracked and clean versions of updated documents should be provided when responding to a provisional approval.

Timeline for providing further information

You have 90 days to provide this further information. Your application will be considered to have been withdrawn if this information is not received on or before 05 July 2021. A new application would be required in this case.

Timeline for giving a final decision

The 35-day clock within which a final decision must be made on this study is suspended as of the date of this letter. This clock, on which 2 days remain, will restart on the date on which **all** of the further information requested above is received by the Northern A Health and Disability Ethics Committee.

How to respond to a Provisional Approval

You will need to submit your new or amended documents through Online Forms.

New versions of existing documents:

Steps	Screenshots
1. Go to the Documents Tab to upload the revised documentation requested by the secretariat	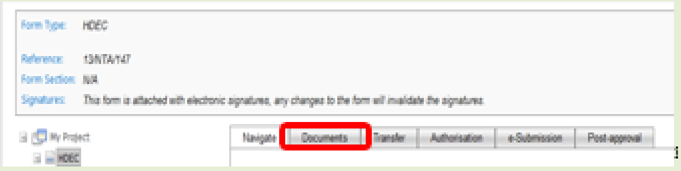
2. To update versions of documents, go to the List tab. Select View/Manage to upload a newer version of the document.<br>• For example you can upload new versions of the PIS/CF<br>• Remember to track changes.	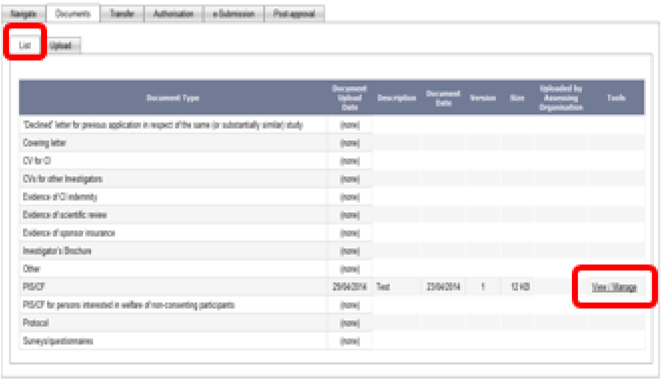
3. When you click View/Manage for a particular document it will take you to the upload tab for that document.<br>• Update the version number and document date.<br>• Browse to find the new version of the file.<br>• Click ‘Upload New Version’<br>• Once the upload is complete the history will populate with the new version.	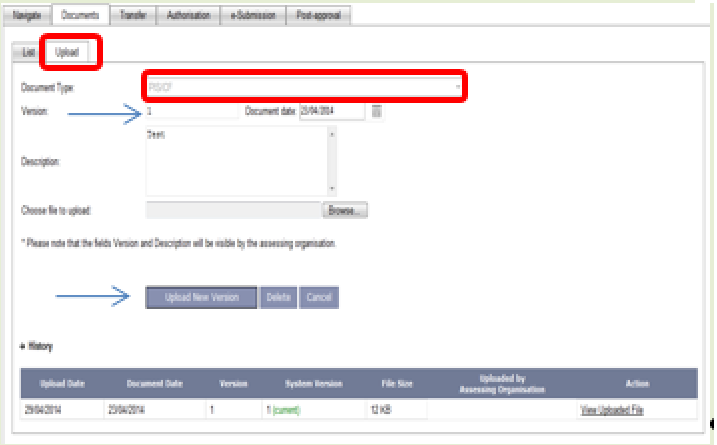<br>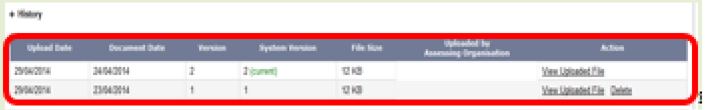

New documents:

Steps	Screenshots

4. For New documents, go to the upload tab.

- For example you can upload a word document responding to questions raised by the Committee.

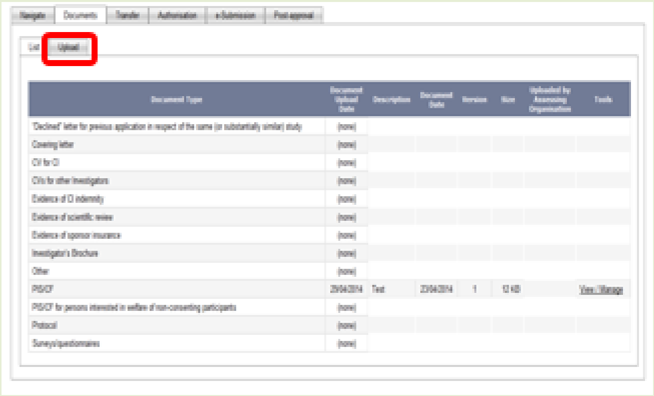

5. Select the document type. Add a version number, document date and add a description if required.

   Browse your computer to find the new file and select Upload File.

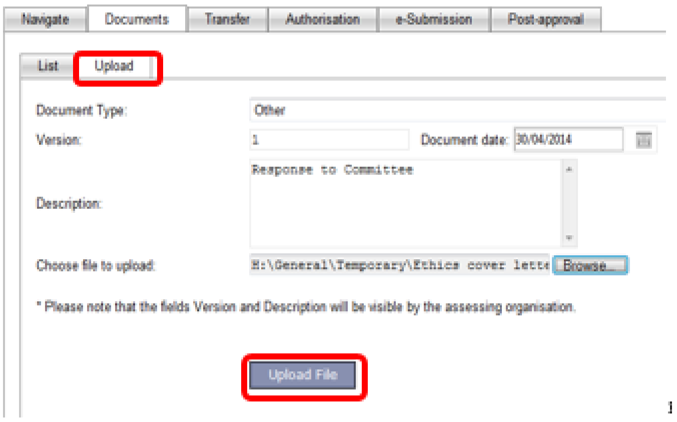

6. The new document will now be uploaded and visible on the List Tab.

   Before submitting check to see all your documents are on the List tab and are displaying the correct version and document date.

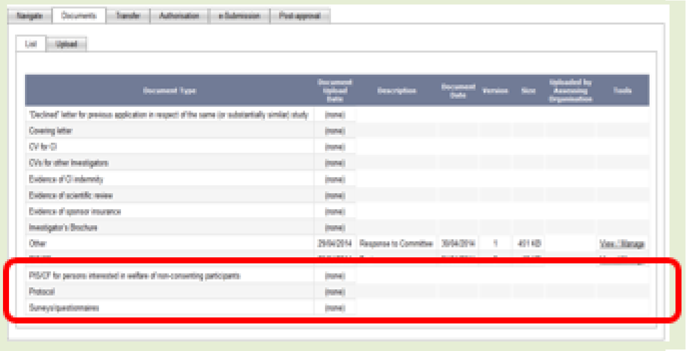

To submit:

7. Once you have uploaded all new documents or updated all existing documents click the E-Submissions tab.	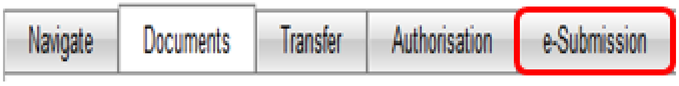
8. Scroll down until you see 'Provisional Approval Response'.<br>This button will only be able to be used when you have received a 'Provisional Approval' letter.<br>**Please note:** only click submit once.	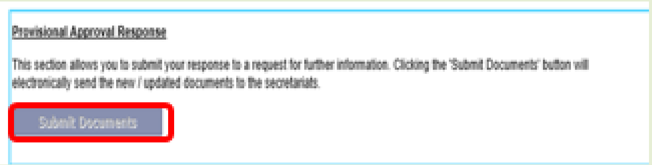

Please don’t hesitate to contact the HDEC secretariat if you have any queries. We look forward to receiving your response.

Yours sincerely,

Mrs Kate O'Connor
Chairperson
Northern A Health and Disability Ethics Committee

Encl:	appendix A:	documents submitted
	appendix B:	statement of compliance and list of members

Appendix A
Documents submitted

Document	*Version*	*Date*
Protocol: This document outlines the protocol for Match Emoji.	Word Document	23 February 2021
CV for CI: CV for Russell Pine	Word Document	23 February 2021
Evidence of scientific review: Aims to articualte the work we have previously completed to inform the design of the game and if the idea was worth further invetigation	Word Document	03 August 2020
Evidence of scientific review: Input from teachers and health professionals into the game.	Word Document	01 September 2020
Evidence of scientific review: Inital systematic review of casual video games.	Word Document	03 February 2020
PIS/CF: Information sheet and ascent form for particiapants	Word Document	23 February 2021
PIS/CF for persons interested in welfare of non-consenting participant: Information sheet and consent for principals	Word Document	23 February 2021
PIS/CF for persons interested in welfare of non-consenting participant: Information sheet and consent form for parent or guardian.	Word Document	23 February 2021
Successful Cure Kids Grant	Email	10 December 2020
HRC Application	Word Document	03 August 2020
Application		25 February 2021
Survey/questionnaire		
Survey/questionnaire		
Survey/questionnaire		
Survey/questionnaire		
Survey/questionnaire		
Other (No Description Entered)		
Other (No Description Entered)		
Other (No Description Entered)		
Other (No Description Entered)		
Other (No Description Entered)		
Other (No Description Entered)		
Other (No Description Entered)		

Appendix B
Statement of compliance and list of members

Statement of compliance

The Northern A Health and Disability Ethics Committee:

- is constituted in accordance with its Terms of Reference
- operates in accordance with the *Standard Operating Procedures for Health and Disability Ethics Committees*, and with the principles of international good clinical practice (GCP)
- is approved by the Health Research Council of New Zealand's Ethics Committee for the purposes of section 25(1)(c) of the Health Research Council Act 1990
- is registered (number 00008714) with the US Department of Health and Human Services' Office for Human Research Protection (OHRP).

List of members

Name	*Category*	*Appointed*	*Term Expires*	*Present on 16/03/2021?*	*Declaration of interest?*
Mrs Kate O'Connor	Lay (consumer/community perspectives)	29/01/2020	29/01/2021	Yes	No
Dr Karen Bartholomew	Non-lay (intervention studies)	18/07/2016	18/07/2022	No	No
Dr Sotera Catapang	Non-lay (observational studies)	11/02/2020	11/02/2023	Yes	No
Ms Catherine Garvey	Lay (the law)	19/03/2019	19/03/2022	Yes	No
Dr Michael Meyer	Non-lay (health/disability service provision)	11/02/2020	11/02/2023	Yes	No
Dr Kate Parker	Non-lay (observational studies)	11/02/2020	11/02/2026	Yes	No
Ms Rochelle Style	Lay (ethical/moral reasoning)	14/06/2017	14/06/2020	Yes	No

Unless members resign, vacate or are removed from their office, every member of HDEC shall continue in office until their successor comes into office (HDEC Terms of Reference)

http://www.ethics.health.govt.nz
