# Supplementary material for: A Casual Video Game With Psychological Well-being Concepts for Young Adolescents: Protocol for an Acceptability and Feasibility Study
Source: JMIR Res Protoc. 2021 Aug 12;10(8):e31588. doi: 10.2196/31588 (PMC8391754; doi:10.2196/31588)
Supplement: Multimedia Appendix 2 [file resprot_v10i8e31588_app2.pdf]

Health and Disability Ethics Committees
Ministry of Health
C/- MEDSAFE, Level 6, Deloitte House
10 Brandon Street
PO Box 5013
Wellington
6011

0800 4 ETHICS
hdecs@moh.govt.nz

SCIENTIFIC PEER REVIEW TEMPLATES
Health and Disability Ethics Committees

INTRODUCTION

The following templates are intended as guidance for researchers who are submitting applications for HDEC review.

The reviewer templates listed below may be used or adapted to suit the particular circumstances. However, please keep within the NEAC informed peer review standards described in the guidelines and in the instruction to researchers and reviewers.

For more information on peer review please refer to Chapter 9 of the National Ethical Standards for Health and Disability Research and Quality Improvement.

SCIENTIFIC PEER REVIEW: Instructions to the researcher

Introduction
Scientific peer review (hereafter referred to as peer review) in the context of human research refers to the scientific validity of the research project and is a vital step in research project development. Peer review is a requirement of ethical approval and can enhance research project development in a variety of ways through providing an objective perspective from an informed reader.

It is a requirement of the Health and Disability Ethics Committees that all research projects involving humans undergo peer review.

Standards for peer review
Peer reviewers will consider the following points in order to determine scientific validity. Your proposal/application should ensure these are addressed.

1. ***The relative merit of the research***: consideration of whether the proposed work is important, worthwhile and justifiable. The research should address a health issue that is important for health and/or society. The aims, research questions and hypotheses should build on and address gaps in existing knowledge.

2. ***The design and methods:*** consideration of the quality of study design and the robustness of the methods used. This might include study methodology, a description of sample recruitment and characteristics (including number, gender and ethnicity where relevant) and proposed methods of data analysis. An indication of timelines for the research should be included.

3. ***The feasibility of the research:*** consideration of whether the overall strategy, methodology and analyses are well reasoned and appropriate to achieve the specific aims of the project. The review will determine whether the research has the likelihood, on balance, of improving scientific knowledge, concepts, technical capacity or methods in the research field, or of contributing to better treatments, services, health outcomes or preventive interventions. The research should be achievable within the specified timeframe and the researcher/research team must have the appropriate experience and expertise to undertake the research.
4. ***Peer review delivers an informed opinion:*** An effective peer review process provides perspectives from subject matter experts. It may be suitable for informed perspectives to be sought from individuals in the same organisation as the researcher, as long as the requirements of freedom from bias, equity and fairness can be met. An appropriate peer is one who can deliver an informed opinion on some or all of a proposal. Reviewers will be knowledgeable about the topic and/or context for the research, have the appropriate expertise relative to the breadth and scope of research under review and, as a result, will be well placed to make a statement as to whether the research in question has

verifiable scientific merit. Peer review of scientific validity may include consideration of cultural relevance and appropriateness.

5. ***Peer review delivers an objective opinion:*** Those acting in the capacity of reviewers are charged with delivering a balanced and considered analysis of the research. Generally, the success of the peer review process is determined by the extent to which these evaluations can be considered free of bias, equitable and fair. Objectivity can be compromised if peer reviewers have conflicts of interest, and so appropriate peer reviewers typically will not be materially connected to the researcher(s) in a way that might undermine objectivity, and be free from either positive or negative inducements.

6. ***A consensus opinion on scientific validity is formed:*** An HDEC will need to receive assurance that the peer review process has delivered support for the scientific validity of the proposed research. When a peer review process has engaged a range of experts, there needs to be a process that leads to a consensus opinion about the quality of the research.

SCIENTIFIC PEER REVIEW:

Date 6th May 2021

Research Title: The feasibility and acceptability of a casual video game with psychological wellbeing concepts for young adolescents

Co-coordinating Investigator: Russell Pine

Peer Reviewer Name: Helen Andreae

Peer Reviewer Position: Lecturer

Independent from study?: Yes

Peer Reviewer signature :Helen Andreae

Recommendation: Approve

REVIEW GUIDELINE	GUIDELINE PROMPTS	COMMENTS
Relative merit of the research	• Important, worthwhile and justifiable.<br>• Addresses a health issue that is important for health and/or society.<br>• Aims, research questions and hypotheses build on and address gaps in existing knowledge.	Resources for supporting mental health are stretched and it is important that we start looking for a range of ways we can support the psychological wellbeing of society. This research is a first step in exploring how digital interaction maybe able to help young people.<br><br>Because this a reasonably new area of research it is good to see that their aims are not over reaching, and that they are choosing to do a feasibility study to set the ground work for those that follow.
Design and methods	• Quality of study design<br>• Robustness of the methods used.<br>• Includes a description of sample recruitment and characteristics (including number, gender and ethnicity where relevant) proposed methods of data analysis.<br>• Timelines for the research included	That they are approaching the research with opened ended questions and are using a range of measures indicates that they are, importantly, not asserting assumptions that they don't yet have evidence for.<br><br>The approach to recruitment was well thought out and had appropriate strategies for addressing participants who disclose mental health needs which require support.<br><br>The measures are appropriate to gage whether their approach and the application is fitting for further research. It is good that both qualitative and quantitative user analytics are being gathered. This will help the researchers understand the links between the analytics and subjective experiences, in order develop more focused and robust measures for follow-up studies.
Feasibility of the research	• Overall strategy, methodology and analyses are well reasoned and appropriate to achieve the specific aims of the project.	The study design and motivation for the research were convincingly argued and will add to the fields understanding of how to test digital applications like match emoji. It will set the ground work to better understand tools for the digital mental health space. Particularly for youth, where there is a knowledge gap.

	• Likely to improve scientific knowledge, concepts, technical capacity or methods in the research field, or of contributing to better treatments, services, health outcomes or preventive interventions.<br>• Achievable within the specified timeframe<br>• Researcher/research team has the appropriate experience and expertise.	The team involved is suitably qualified to carry out this research and have a diverse and complimentary range of skills and experience. Their combined perspectives will add to the robustness of the research outputs and gives me confidence they have the skills to carry out this research and work with youth.
Reviewer Independence /objectivity	• Peer review is considered free of bias, equitable and fair.<br>• Objectivity can be compromised if peer reviewers have conflicts of interest, and so appropriate peer reviewers typically will not be materially connected to the researcher(s) in a way that might undermine objectivity, and be free from either positive or negative inducements.<br>• If the peer reviewer is connected to the study please explain what measures are taken to mitigate conflict of interest.	I'm not connected with the study and anyway and have no material interest in the outcome of the study. I have worked with some of the researchers on other projects but this research is not tied to that.
Other comments	• Any reviewer observations that are not covered in the points above.	
